# Shrimp Farming Practices in the Puttallam District of Sri Lanka: Implications for Disease Control, Industry Sustainability, and Rural Development

**DOI:** 10.4061/2010/679130

**Published:** 2010-08-12

**Authors:** M. Nalaka Munasinghe, Craig Stephen, Preeni Abeynayake, Indra S. Abeygunawardena

**Affiliations:** ^1^Faculty of Veterinary Medicine and Animal Science, University of Peradeniya, Peradeniya 20400, Sri Lanka; ^2^Faculty of Veterinary Medicine, University of Calgary, 3280 Hospital Drive NW., Calgary, AB, Canada T2N 2Z6; ^3^Centre for Coastal Health. Nanaimo, 900 5th Street, BC, Canada V9R 5S5

## Abstract

Shrimp farming has great potential to diversify and secure income in rural Sri Lanka, but production has significantly declined in recent years due to civil conflicts, some unsustainable practices and devastating outbreaks of disease. We examined management practices affecting disease prevention and control in the Puttalam district to identify extension services outputs that could support sustainable development of Sri Lankan shrimp farming. A survey on 621 shrimp farms (603 operational and 18 nonoperational) was conducted within the Puttalam district over 42 weeks comprising a series of three-day field visits from August 2008 to October 2009, covering two consecutive shrimp crops. Fundamental deficits in disease control, management, and biosecurity practices were found. Farmers had knowledge of biosecurity but the lack of financial resources was a major impediment to improved disease control. Smallholder farmers were disproportionately constrained in their ability to enact basic biosecurity practices due to their economic status. Basic breaches in biosecurity will keep disease as the rate limiting step in this industry. Plans to support this industry must recognize the socioeconomic reality of rural Sri Lankan aquaculture.

## 1. Introduction

Farmed shrimp export accounts for approximately 50% of the total export earnings from Sri Lankan fisheries [1]. It was their second most valuable export fisheries in 2007, generating Rs2487 millions (approx 25 million USD) [2]. More than 90% of the harvested cultured shrimp are exported, going mostly to Japan followed by United States of America and countries of European Union [1]. The black tiger shrimp, *Penaus monodon* is the main species cultured. The majority of grow out shrimp farms in Sri Lanka follows semi-intensive culture practice [3]. Farmed shrimp production was 2,220 mt in 2008 compared to 9,240 mt from wild capture [4]. Aquaculture production peaked in 1998 at 6,520 mt. The shrimp industry was responsible for 40,000 direct and indirect jobs in 1996 [5] representing 11% of the total employment in the fisheries sector [6]. More recent estimates report that jobs have declined to 8,000 due to contraction of the industry in large part because of the effects of disease outbreaks [1]. The potential for this industry to once again provide a large number of jobs and export income makes its development very attractive to the government of Sri Lanka. 

**Figure 1:**
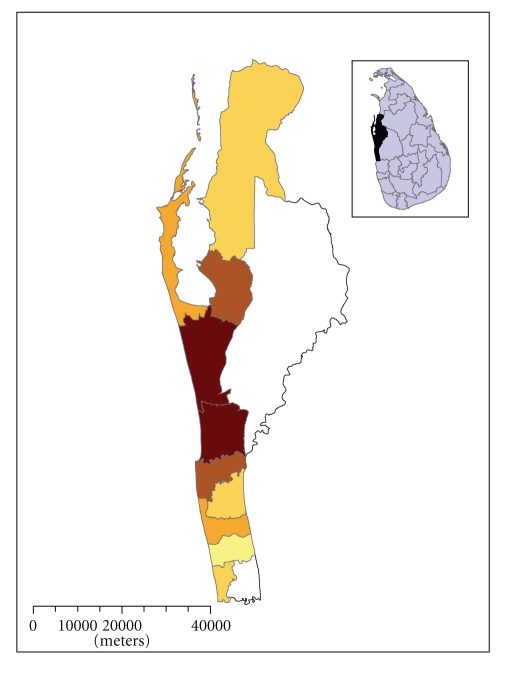
Shrimp farming zones of the Puttalam district, Sri Lanka. Shading reflects farm density with darker colours being higher density. Insert figure is a map of Sri Lanka which locates the Puttalam district.

Shrimp farming was begun on the eastern coast in the Batticaloa District in the late 1970s but was abandoned due to civil unrest [7]. The industry was revived during the early 1980s along the coastal border of the Puttalam district of the North Western region of Sri Lanka (Figure 1). The industry rapidly expanded in the Puttalam district for three major reasons. First, there were abundant natural resources such as coastal lagoons, mangrove swamps, tidal flats and, estuaries well-suited to shrimp farming. Second, this region had a good road network and easy access to the Katunayake international airport and Colombo harbor which allowed for quick access to infrastructure needs for the export of fresh product. Third, industry development in the Puttalam district coincided with a heavy demand for shrimp in international markets. 

Industry growth was slowed in the 1990, in large part due to the epizootic viral diseases Monodon Baculo Virus (MBv) and white spot syndrome virus (WSSv) [5]. WSSv was first reported in 1996. MBv was first found in Sri Lanka in 1988 in imported post larval (PL) stocks from Thailand. Losses due to white spot disease (WSD) were valued at Rs1 billion [6]. Yellow head disease (YHD) was recognized in Sri Lanka in 1998 in infected brood stocks. The dual problems of WSD and YHD caused an approximate 70% drop in exported shrimp products [6]. Illegal use of state lands, mangrove habitat destruction and poor farm construction lead to a variety of environmental and socioeconomic effects that further impacted the growth and sustainability of shrimp farming in Puttalam [3]. The industry has still not recovered from these impacts and thus its promises as a means for rural economic development have been left unfulfilled. With the end of the civil war in 2009, Sri Lanka began to look for means to revive and expand this industry to supports its goals for rural development and economic growth in the north and east of the country. 

Prevention and control of disease in farmed shrimp is a priority for the growth and sustainability of this industry for two main reasons: First, to maintain consumer confidence. There is increasing public concern about the use of antimicrobial drugs and chemicals in shrimp farms [8]. Some have suggested insufficient understanding of appropriate antimicrobial use coupled with an inadequate legislative framework has lead to misuse of antimicrobials in many shrimp farming countries [8]. This perception may threaten industry sustainability due to its impacts on consumer confidence. Reducing the need for antimicrobials through reduced rates and impacts of disease is a key to removing this threat. Second, disease outbreaks continue to directly interrupt the growth and viability of the industry through shrimp morbidity and mortality despite the advances in biosecurity knowledge and the delivery of training programs, seminars and workshops to farmers to transfer new technology. There has not been a systematic evaluation of biosecurity and disease management on Sri Lankan shrimp farms even though there is international recognition that disease control is an industry priority. Our objective was to investigate shrimp farming practices in the Puttalam district that may affect disease prevention and control in order to identify targets for extension services that could support the sustainability and expansion of a safe industry in Sri Lanka. 

## 2. Methodology

A list of licensed operating and nonoperational shrimp farms was obtained from the National Aquaculture Development Authority (NAQDA). Of the 1176 farms identified by NAQDA, 603 were recorded to be in operation. We undertook a complete survey of 621 farms within the Puttalam district over 42 weeks, including all of the operating farms and 18 abandoned or nonoperating farms. A series of three-day field visits were conducted from August 2008 to October 2009 covering two consecutive shrimp crops. Five hundred and sixteen people, of them 383 owners, 46 managers, 83 supervisors, and 11 other people, were interviewed using a standard questionnaire. Questions focused on general management practices, health management and control points for biosecurity. Areas covered included farm infrastructure; water sources and pond management; movements of people, animals, and feed; use and access to health services; use and access to vaccines, medications, or other compounds used to prevent or treat disease; sources of stock; and production costs. Participation of farmers and staff was voluntary. The interviews were supplemented with on-farm veterinary visits aimed at collecting observation of the farming sites and operations as well as the surrounding environment. All data were collected in an anonymous manner and entered into Microsoft Access. This research was conducted after approval from the University of Calgary Conjoint Faculties Research Ethics Board and ethical review by the Faculty of Veterinary Medicine and Animal Science, University of Peradeniya. 

## 3. Results

The total number of licensed farms (operating and non-operating) reported by NAQDA was an underestimate. We found 2536 available ponds with 1885 (74%) operating. Some of this difference was explained by the practice of sub-dividing licensed farms. The 603 licensed operating farms occupied 1404.6 ha. Most of the abandoned farms were permanently abandoned and converted to salt production, with only a few being used intermittently for farming. Shrimp farming was practiced in nine divisional secretariat divisions (DSDs), but the majority of operating farms (including the highest proportion of large farms) were concentrated in two DSDs, namely, Mundalama (*n* = 221; 44 farms >2–5 ha) and Arachchikattuwa (*n* = 256; 24 farms >2–5 ha). The Mundalama DSDs was the largest shrimp farming area occupying 452 hectares. Shrimp farming areas in the Putallam district were administratively divided by government into 34 subzones. The designation of these sub-zones did not consistently reflect biological considerations, such as shared water sources. Sometimes, physical structures, such as roads, separated zones. Fifty-four percent of farms of the Puttalam district were less than 1 hectare and 73% of farms were less than 2 ha. The functioning large scale farms of >5 ha represented 9% of surveyed farms with the remaining 18% being between 2 and 5 ha. Family operated small scale farms of 0.25–1 ha were most common in 6 subzones (Pinkattiya, Muthupanthiya, Udappuwa, Kottage, Naguleliya, and Pulichchankulama). Most of the family members of these regions were involved in shrimp farming and used it as their major source of income. Many of these farmers rented their ponds and relied on loans or pawning personal possessions to purchase PL and feed. These farmers could not afford hired labor. 

The cost of production had more than doubled in the past decade while the purchase price paid to farmers had almost halved. For example diesel rose from approx Rs30/l to Rs70–110/l; feed from Rs50/kg to Rs 200/kg and labour from Rs4000/mos to Rs8000–12000/mos. Over the same period, the sale price has declined from Rs900/kg to Rs450–600/kg. Power was an important cost to farmers. For example, on a farm that used 900 paddle wheel hours for aeration and 100 hours of water pumping, 2010 diesel costs could be Rs30–70/kg shrimp produced, resulting in some large farms spending more than Rs40,000/day on diesel. Electricity was less expensive than diesel or kerosene but had an initial investment for infrastructure that could make it an unaffordable alternative, especially to small scale family farms. Many farms of medium to large scale which did not have electricity had closed due to the high fuel cost. 

Economic pressures lead to increasing numbers of farmers harvesting at 3–3.5 months for the domestic market. The usual production target was 30 g shrimp within 4 month of pond culture. However, when the market price was favorable, many farms did a partial harvest after the 3rd month when an average shrimp weight was 20 g. In recent years, the lower export price and reasonable local market price caused many farmers to sell their harvest to the local market. At the time of this survey, the price paid for 1 kg of 30 g shrimp to farmers was Rs450–600. Farmers received approximately Rs500/kg for 20 g shrimp in the domestic market. Competition did little to affect the price due to domination of the export market by a small number of exporters. During the time of our survey, 197 farms (33%) shifted to this shorter production cycle. Forty percent (*n* = 21) of the 53 large farms and 19 of the 222 medium-sized farms (9.6%) had reduced the scale of production. Some large scale farms were not putting all of their available ponds into production, especially in Madampe, Madurankuliya, Bangadeniya, and Anakutti divisions. Others stocked their ponds only for one of the two possible crop cycles per year. 

Farmer complained about poor shrimp growth rates in recent years and attributed this to poor PL or feed quality, although there has been no investigation of its cause. They also reported increase variation in body size and weight of shrimp at harvest. The poor weight gains resulted in lengthening the crop period, increasing costs and further lowering the profit margin. Some farmers were shifting to lower stocking densities to reduce feed costs and labor costs associated with the needs for water changes and aeration. 

The total brackish water resources of the Puttalam district consists of 44844 ha of lagoons, salt marshes, and mangrove forests [4]. The principle water resources were the Puttalama lagoon, Mundalama lagoon, and Chilaw lagoon. The Dutch Canal interconnects these lagoons with the Negombo lagoon and the Daduru oya and Mi oya river basins which bring averages of 1129 million m^3^ and 198 million m^3^of water, respectively, annually to the district [9]. Sixty-eight percent of total number of operating farms we identified was located on the Mundalama lagoon and the Dutch Canal. Senarath, 1998 [10] reported that farmers commonly used stocking rates of 5–12 PL/m^2^ for farms without aeration and 12–25 PL/m^2^on farms with paddle wheel aeration: we found farmers stocking 7.5–12.5 PL/m^2^ on farms without paddle wheel aeration to reduce the production cost.

Fresh water input to the three lagoons increased during the rainy season of November to December and caused local flooding. Many roads in the area disappeared or became damaged due to the rising water level creating difficulties in transporting crops, feed, and personnel. Flooding altered water quality, in particular it changed salinity, alkalinity and turbidity. Water inlets and outlets merged as water levels rose in the Dutch Canal. Lowered salinity most markedly affected farms along the river basins from Mundalama lagoon to the southern end of the coastal belt of the Puttalam district. During the rainy period, farmers had to postpone stocking until salinity levels returned to normal, usually within 2 weeks after the rains stopped. Removal of sand in the Thoduwawa estuary was an engineering solution that was providing an influx of saline tidal water into the lagoons. 

Large scale farms consumed large quantity of water during the dry period reducing the amount of water available to smaller family farms and the local community. Engineering solutions that will make more fresh water available were being undertaken to reduce this problem. During the late dry period from August to September, the salinity levels exceeded 50ppt in brackish water around the Puttalama district. This is substantially higher than salinity for optimal shrimp growth, which varies by species but is typically <30ppt and for *P. monodon* can be closer to freshwater-brackish water values [11]. Many farmers had constructed tube wells as fresh water sources for use in this period. Stocking times were adjusted to address delayed harvests, lack of PL and diseases.

Ninety percent of farms (*n* = 545) practiced open system culture with direct pumping of water to the ponds. The majority (*n* = 469, 78%) obtained water out of canals that connected to the water source with the remainder (22%) pumping water directly from the water source. The construction and connections of the canals was complex in community farm regions where family farms were subdivisions of larger farms. The remainder, consisting of medium and large scale farms, used a semiclosed system of water management. Semi-closed systems had separate water intakes and outlets and had a stock tank into which water could be pumped and treated before entering ponds. Only 9.6% (58) of farms used stock tanks, which are spare tanks used to hold water in reserve for later use. Stock tanks served as water sources to replace evaporated water from the pond and for water changes. Even though 23% of farms had abandoned ponds, most farmers did not maintain stock tanks due to added costs of diesel to operate the pumps. Semi-closed operations could temporarily close the system by stopping water change. They maintained water levels by additions from the stock tank. This method was used during disease outbreaks, however, the high biomass of high-density farms and increasing biomass due to the growth rapidly degraded water quality. 

Farmers using stock tanks could treat the water before using it in their ponds in semiclosed systems but 78% of farms did not use any chemical disinfection, largely due to economic constraints. Eighty-four farms (14%) used chlorine as disinfectant solution 49 (8%) farms used pesticides and 12 (2%) used both. Treatments were applied after the ponds were filled and kept empty of PL for 7 days for chlorine and 15 days for pesticides. The principle pesticide found was (RS)-œ-cyano-3-phenoxybenzyl (1RS, 3RS; 1RS,3RS)-3-2,2-dichlorovinyl)-2,2-dimethylcyclopropane carboxylate 25%. Application rates varied with brand name. 

Only 90 farms (14.9%) used nets for filtering incoming water. The small-scale farms that filtered water, largely restricted filtration to the beginning of the culture period. Few farms (*n* = 52, 9%) used filter nets throughout the culture period. Growth of small crustaceans and fish were observed in farms not practicing adequate water filtration. Twenty-five percent (*n* = 148) of farmers used tea seeds to destroy fish in ponds after 2.5 months. Neither open nor semi-closed systems treated effluent water before release. Medium-to large-scale farms tended to have better separated inlets for influent water and long outlet canals that allowed sedimentation of outflow water. Although some farmers tried to maintain separate water inlets and outlets, they ultimately mixed in the water source. Irregular and sometime unapproved constructions of ponds and water canals caused difficulties in separating incoming and outgoing water, especially in family and small-scale farms. The density of farms in regions with predominately small scale farms was 1.3–1.6 farms/ha. 

Pond water pH, alkalinity, and salinity were checked once a week by the consultants from feed and chemical supply companies. Only 7 small-scale farmers reported that this service was not available to them. Consulting service providers measured dissolved oxygen, turbidity, pH, and salinity routinely on 596 farms, but ammonia was only checked when there were signs of problems in the shrimp. Ammonia problems were typically not encountered until the 3rd month of production. Oxytetracycline was used on 15% (92/603) of the functioning farms. Farmers added commercially prepared probiotics to ponds to combat what they call “white fecal syndrome” which they attribute to environmental bacteria in the ponds. 

Water changes typically started after 30–45 days of PL stocking in 81% of farms while 9% of farms started to change water before day 30. Farmers typically changed 15–45 cm of water for ponds that were 100–140 cm deep. Occasionally, farmers changed 1/3 to 2/3 of the water in a pond. The amount of the water changed and frequency of changes increased through the production cycle, water changes were done once or twice a week at the start but increased to every day or every other day near the end of production. Amounts and frequencies of water changes were altered to address water quality issues. Sometimes, water changes could not be done in areas where family farms were densely packed, when there was a harvest, or when water change took place in the adjacent farm. Paddle wheels were the main method of aeration on farms using aeration. Farmers usually used 4 paddle wheels per 0.4 ha of pond-for 4–18 hours. Aeration increased with the increasing age of the shrimp, on cloudy and less windy days and at night. 

Ponds were built with mud banks and mud floor. The stocking seasonality was planned by NAQDA to achieve a minimum of 2 month period between two consecutive crops to allow the ponds to dry. Pond bottoms were dried until cracks appeared in the sediment layer. This kind of drying could not be attained during the rainy season from October to January. The seasonal crop calendar was prepared by getting comments from a technical committee comprised of experienced farmers representing farmer societies of different farming zones and NAQDA extension officers. Each subzone was assigned a period of time for stocking shrimp. There were two seasons of shrimp culture. This period varied with weather, delays in harvests, and disease outbreaks. 

Of the 603 functioning farms, 13 completely removed organic wastes from the ponds. The rest used the organic waste from pond bottoms to repair their pond banks. They did not remove the cracked layer properly during pond preparation. Farmers removed the wastes using machinery or manual labors. Harrowing of the pond bottom was seen in some farms especially those with sand bottoms. Lime and/or dolomite was used on 481 farms (80%), 44 (7%) used dolomite only, 7 (1%) used lime only, and the remainder (12%) used neither. Although the main objective of these chemical applications was to correct the pond bottom pH, only 21% (*n* = 129) of farmers checked soil pH or calculated the amount of lime and dolomite to be applied. The amount applied depended on the farmers' experience and economic factors. Fertilizers were used by 214 (35.5%) of farms; most commonly used fertilizers were untreated cattle dung (73/214) and poultry litter (15/214). 

None of the farms had vehicle tire baths at the entrance. Shrimp farmers often also reared or owned cattle, goats, birds, and some companion animals. Most farms (*n* = 501, 83%) lacked outer fences, thus allowing open access to people and animals which we observed walking on the pond banks on several occasions. Only 101 (17%) of farms had barb wire fences but they tended to be poorly constructed and covered only one or two sides of the farm, with water canals, jungle, or other farms acting as barriers on the remaining sides. Some farmers complain that they were robbed of shrimp at night during the later stage of the crop and complained about their fences being cut. No one was often present on small-scale farms during the day, except for feeding times because the farmers tended to rest during the day as they were up at night guarding their farms and they could not hire staff. 

The little cormorant is a troublesome predator on Sri Lankan shrimp farms but only 25% of farms (*n* = 147) had proper bird nets. Small scale family farms generally lacked bird netting over ponds. The additional cost of installing the nets was cited as a disincentive for their use. We saw many attempts to place one or two plastic strips across ponds to discourage birds, but these were often too far apart or too few to be effective deterrents.

All farms used commercially prepared feeds imported from Thailand. Feed or feed supplements were not screened for pathogens by the farmer or any regulating body before delivery to the farms. Feeding frequency varied from three times/day at the beginning of production to five times/day a day at the end. The amount of feed provided increased with the age of shrimp but was usually measured by observing the consumption of feed in the feed tray within two hours after feeding. A variety of proprietary vitamin, mineral, and probiotic mixes were added to the feed in the second month of production. Probiotics and, vitamins were used in 217 farms (36%), probiotics, vitamins and minerals on 9 farms (1.5%), and probiotics and minerals on 10 farms (1.7%). 

Forty-one farms (6.8%) used wild captured shellfish and 46 (7.6%) used wild caught fish as a protein supplement to try to compensate for the poor growth rates they had been experiencing. Fish was used more at the beginning of the culture period and clams were usually fed daily in the last one or two weeks of culture. The fish and clams were usually, but not always, cooked or boiled. Raw chicken eggs were used as a binder for feed additives at 190 farms (31.5%). 

Health management services and support were few. NAQDA was the principle public sector source. The agency undertook broodstock screening, monitored biosecurity practices, and provided some diagnostic services, but the latter was limited to tests for a small number of pathogens. Broodstock came from the wild capture and were shipped to hatcheries. The hatcheries produce 15-day old PL that were conditioned to the desired salinity level and shipped to the farms. Regulations required the PL stock in the hatchery be checked for MBv and WSSv before they were to be distributed to grow out sites and results reported to NAQDA. Hatchery owners would pay private laboratories approximately Rs5,000 for this service. Shrimp in grow out ponds were not tested to detect viral threats during the culture period. If a farmer complained about shrimp death to NAQDA, the agency was generally limited to testing for MBv and WSSv. Once a disease outbreak was noticed, the affected ponds were destroyed by NAQDA using pesticides as a disease controlling strategy. Sometimes, they also delayed PL stocking for the next crop in affected sub zones. Hatcheries were required to provide certificates of WSSv and MBv free status from NAQDA before shipping PL to farms. However, many farmers complained that they had to face bad responses from the hatchery owners when they requested that PL be checked for diseases and, therefore, some farmers stocked PL without WSSv or MBv screening. Farmers did not disinfect PL or PL containers before putting them into the ponds.

There were no veterinary services available to farmers. Private laboratory facilities were available but were reported to usually be too expensive. Feed and chemical supply companies provided some free consultation in water quality and farm management and were an important source of health extension services to farmers. Some farmers, however, were cautious of the use of these representatives because of the conflicts between providing health advice and selling specific products and they questioned the background or training of some consultants. Farmer societies were present in each sub-zone and they had some responsibility for monitoring biosecurity efforts. 

## 4. Conclusion

Sri Lanka is a rural-based society which is looking to aquaculture to diversify rural incomes and reduce rural-urban migration. For example, its fisheries development plan for the northern province, where the fisheries sector collapsed due to the past civil war and the tsunami of 2004, includes an important role of aquaculture development. However, the current disease control and prevention practices in the Puttalam district were incompatible with the goal of reducing disease as an impediment to industry sustainability and if not remedied, would likely prevent successful expansion of the industry to other parts of the country. Shrimp disease has previously been associated not only with reduced farm productivity but also with impacts on rural community sustainability [12]. Smallholder farmers were disproportionately constrained in their ability to enact basic biosecurity practices due to their economic realities. Rural development built upon smallholder agriculture economy is widely seen as the cornerstone of poverty reduction [13]. Aquaculture has become or has the potential to become an attractive and important component of this economy. Unfortunately, the development of aquaculture in low and middle income countries has not been without its problems. Exclusion of small-scale farmers, environmental degradation, and disease outbreaks have lead to criticisms of aquaculture as a means for rural development. 

One of the recommendations from the World Wildlife Funds Shrimp Dialogue is for shrimp farms to;** “**Adopt health management plans that aim to reduce stress, minimize the risk of diseases affecting the cultured and wild stocks, and increase food safety” (http://www.worldwildlife.org/what/globalmarkets/aquaculture/dialogues-shrimp.html). The application of biosecurity measures for aquaculture was discussed first in 1997 in a world aquaculture society special session titled “Sustainable Shrimp Farming: Emerging Technologies and Products for Biosecurity and Zero Discharge” [14]. Thereafter, several workshops were held to clarify the application of biosecurity measures in aquaculture [14]. Many papers outline standards of practice needed to achieve acceptable levels of shrimp farm biosecurity and disease control (e.g., [14–18]). We found fundamental deficits in disease control, management and biosecurity practices on shrimp farms in the Puttalam district of Sri Lanka, despite these recent advances in knowledge and guidelines on shrimp health management. It was evident that the main impediment to improved biosecurity was not necessarily the lack of knowledge, but instead the lack of economically viable means to reach biosecurity goals. Economic issues have reduced shrimp production in the Puttalam district and were reported to significantly restrict farmers' willingness or ability to invest in biosecurity. Aquaculture extension in Sri Lanka will need to recognize this reality and explore low cost ways to improve the capacity for farms, especially smallholder farms, to prevent the introduction of pathogens, detect and respond quickly to disease events, and have effect means for containing disease outbreaks. Without attention to the basic socioeconomic factors influencing farmer practices, the Sri Lankan shrimp farming industry will remain vulnerable to industry limiting disease outbreaks.

Disease is but one factor affecting the growth and sustainability of shrimp farming. Impacts on local water supplies and quality, conflicts with other land users, environmental degradation are key issues that Sri Lanka aquaculture managers will need to addressed if shrimp farming is to become part of a long term development strategy [1, 3]. However, disease control remains the current rate limiting step in this industry which must be addressed if farmers are to realize a profit from their farms that can be turned back into infrastructure, materials or practices that will be more sustainable.
